# Diminished Physical Activity in Older Hospitalised Patients with and without COVID-19

**DOI:** 10.3390/jcm12196261

**Published:** 2023-09-28

**Authors:** Karolina Piotrowicz, Ian Perera, Monika Ryś, Anna Skalska, Suzy V. Hope, Barbara Gryglewska, Jean-Pierre Michel, Tomasz Grodzicki, Jerzy Gąsowski

**Affiliations:** 1 Department of Internal Medicine and Gerontology, Jagiellonian University Medical College, 30-688 Kraków, Poland; 2 University Hospital, 30-688 Kraków, Poland; 3 College of Medicine and Health, University of Exeter, and Royal Devon & Exeter NHS Foundation Trust, Exeter EX2 5DW, UK; 4 University of Geneva, 1205 Geneva, Switzerland

**Keywords:** physical activity, COVID-19, older patients, hospitalised, activPAL^®^

## Abstract

Acute viral respiratory infections have proven to be a major health threat, even after the Corona Virus Disease 2019 (COVID-19) pandemic. We aimed to check whether the presence or absence of an acute respiratory infection such as COVID-19 can influence the physical activity of older hospitalised patients. We cross-sectionally studied patients aged ≥60 years, hospitalized during the pandemic in the non-COVID-19 and COVID-19 ward at the University Hospital, Kraków, Poland. Using activPAL3^®^ technology, we assessed physical activity for 24 h upon admission and discharge. In addition, we applied the sarcopenia screening tool (SARC-F); measured the hand grip strength and calf circumference; and assessed the Modified Early Warning Score (MEWS), age-adjusted Charlson Index, SpO2%, and length of stay (LoS). Data were analysed using SAS 9.4. The mean (min, max) age of the 31 (58% women, eight with COVID-19) consecutive patients was 79.0 (62, 101, respectively) years. The daily time (activPAL3^®^, median [p5, p95], in hours) spent sitting or reclining was 23.7 [17.2, 24] upon admission and 23.5 [17.8, 24] at discharge. The time spent standing was 0.23 [0.0, 5.0] upon admission and 0.4 [0.0, 4.6] at discharge. The corresponding values for walking were 0.0 [0.0, 0.4] and 0.1 [0.0, 0.5]. SARC-F, admission hand grip strength, calf circumference, and LoS were correlated with physical activity upon admission and discharge (all *p* < 0.04). For every unit increase in SARC-F, there was a 0.07 h shorter walking time upon discharge. None of the above results differed between patients with and without COVID-19. The level of physical activity in older patients hospitalised during the pandemic was low, and was dependent on muscular function upon admission but not on COVID-19 status. This has ramifications for scenarios other than pandemic clinical scenarios.

## 1. Introduction

Data published before the COVID-19 pandemic indicate that older inpatients spend do not spend much a lot of time moving about [1,2,3]. In COVID-19, the low level of physical activity in older COVID-19 inpatients was very low indeed, with the potential to promote acute sarcopenia and functional deficits. On the other hand, it is possible that rules about social distancing, fear of infection, and restrictions on entering or leaving patient rooms may have had an impact on the level of physical activity in older non-COVID-19, especially multimorbid, patients who were hospitalised during the pandemic [4]. Such attitudes still linger, especially with challenges such as respiratory syncytial virus (RSV) infections. Clearly, some of the isolation procedures that were developed during the COVID-19 pandemic are currently being adopted in order to mitigate new threats. On the one hand, these measures may help limit the spread of RSV or other respiratory viral infections; on the other hand, these measures could result in restrictions on both spontaneous physical activity and physiotherapeutic activities.

Physical activity is one of the key factors promoting better quality of life and survival in older individuals [5]. Adequate rehabilitation has been shown to improve outcomes in older hospitalised patients [6]. Greater pre-hospitalisation dependence, including less physical activity, has been associated with greater worsening of sarcopenia during acute hospitalisation [7]. Thus, alterations in the level of physical activity can have broad clinical implications in older patients and are worth studying.

Currently, there are no unified guidelines regarding the duration and intensity of in-hospital physical activity for older, acute, especially multimorbid patients, and no such guidelines exist for patients infected with SARS-CoV-2 or RSV [8]. We aimed to assess the time spent participating in physical activity and its associated factors, among older hospitalised patients with and without COVID-19 upon admission and at discharge.

## 2. Methods

In our cross-sectional study, we included consecutive COVID-19 and non-COVID-19 patients aged ≥60 years, who, at the time of the pandemic, were hospitalised at the geriatric unit of the Department of Internal Medicine and Geriatrics, University Hospital, Kraków, Poland, between 27 March and 30 April 2021 (COVID-19 patients, hospitalised in the temporary COVID-19 ward), and 1 May and 30 May 2021 (the non-COVID-19 patients). Using SARC-F, we screened the patients for sarcopenia 14 days prior to hospitalisation [9]. We calculated the age-adjusted Charlson Comorbidity Index (aCCI) [10]. The severity of the patient’s status was assessed using the Modified Early Warning Score (MEWS) inventory [11]. We measured muscular strength at baseline using the European Working Group on Sarcopenia in Older People 2 (EWGSOP2) hand-grip methodology [12]. SpO2% was measured with patients breathing air or receiving oxygen supplementation as needed to reach the optimal SpO2%. Calf circumference was measured as a proxy for muscle mass. The diagnosis of probable sarcopenia was based on the EWGSOP2 criteria [12]. The activPAL3^®^ (Pal Technologies Ltd., Glasgow, UK) activity tracker recorded the patient’s physical activity for an initial 24 h period (during the first 48 h of hospitalisation) and at discharge (within last 48 h of hospitalisation). The device was attached to the mid-portion of the front of the right thigh. For the analysis of the unedited activity data, we used the manufacturer’s definitions and the software included with the device. The approval of the Jagiellonian University Ethics Committee was obtained (decision number 1072.6120.27.2021) and the patients consented to engage in the study. The COVID-19 part of the study was performed in a COVID-19 ward with a high level of protective routines, requiring the use of high-grade airborne infection personal protective equipment (PPE) [13]. The same, standard, rehabilitation model and staff, consisting of three physiotherapists, were involved in the care for all patients. Statistical analysis was performed with the SAS 9.4 (SAS Institute Inc., Cary, NC, USA). For the comparisons, we used the Wilcoxon’s test. The proportions were compared using the Chi-square test. We performed Spearman’s correlation analysis (with Fisher’s transformation—based on 95% confidence intervals) in the entire group and checked the independence of the results in COVID-19 status adjusted and mutually adjusted linear regression. We did not perform an a priori sample-size calculation.

## 3. Results

The mean (min, max) age of the 31 (58% women) patients was 79.0 (62, 101, respectively) years. Eight patients were hospitalised in the COVID-19 ward and the rest were hospitalised in the regular non-COVID-19 ward. The COVID-19 patients had lower aCCI (median 4.5 vs. 6.0, *p =* 0.02). Compared with non-COVID-19 patients, fewer patients with SARS-CoV-2 infection had probable sarcopenia (8 of 23 vs. 3 of 8, *p =* 0.03). The patients with COVID-19 had higher admission MEWS than those without COVID-19 (median 2 vs. 0, *p =* 0.03). The admission SpO2% did not differ between the COVID-19 and the non-COVID-19 patients (median [p5, p95], 94% [88%, 97%] vs. 96% [88%, 98%], *p =* 0.36, COVID-19 and non-COVID-19, respectively). The duration of hospitalisation did not differ between the COVID-19 and non-COVID-19 patients, with a median (min, max) of 11 (4, 44) days.

**Figure 1:**
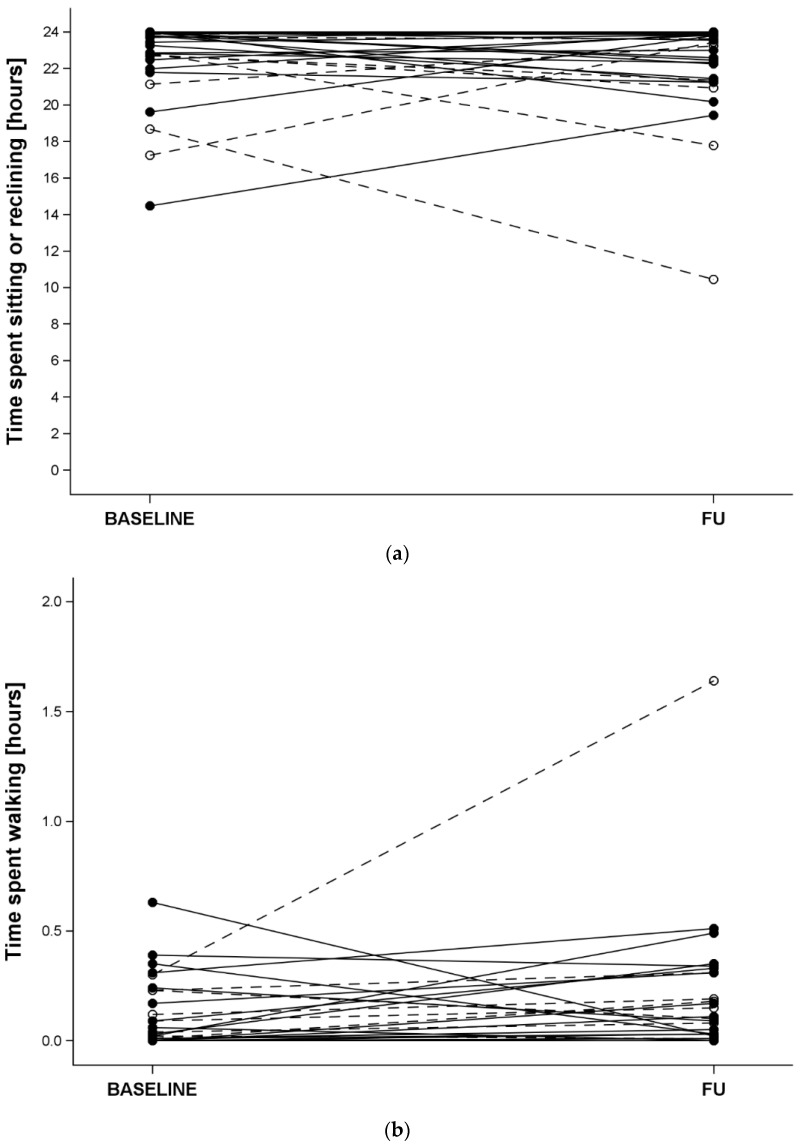
Top panel—time spent sitting or reclining, bottom panel—time spent walking. At admission (BASELINE) and discharge (FU) in COVID-19 (open symbols and dashed lines) and non-COVID-19 (full symbols and solid lines) hospitalised older patients.

Upon admission, the time spent sitting or reclining was less (*p =* 0.02, Figure 1a) and the time spent standing was longer (*p =* 0.04) in COVID-19 than non-COVID-19 patients. Time spent walking did not significantly differ between the two groups (*p =* 0.09, Figure 1b).

The corresponding discharge values and the differences between baseline and follow-up did not differ between the groups (all *p* > 0.10). Overall, the time (median [p5, p95], in hours) spent sitting or reclining upon admission was 23.7 [17.2, 24] and at discharge it was 23.5 [17.8, 24]. The time spent standing upon admission was 0.23 [0.0, 5.0] and at discharge it was 0.4 [0.0, 4.6]. The corresponding values for walking were 0.0 [0.0, 0.4] and 0.1 [0.0, 0.5], respectively.

The results of the correlation analysis are contained in Table 1. Overall, there was no relation between SpO2% or MEWS upon admission and the activPAL3^®^-derived measures of physical activity (all *p* > 0.18). aCCI was correlated with measures of physical activity at discharge (all *p* < 0.06) and time spent sitting or reclining upon admission (*p =* 0.04). The measures of sarcopenia (SARC-F, admission hand grip strength, and calf circumference) were correlated with both admission and discharge measures of physical activity (all *p* < 0.04, Table 1). The linear regression models, which included variables significantly correlated with individual measures of physical activity that were additionally adjusted for COVID-19 status, did not show an influence on COVID-19 status. The mutually adjusted model showed that an increase of 1 unit in SARC-F score was associated with a 0.06 h (beta −0.06, 95% CI −0.13, 0.01, *p* −0.03) shorter discharge walking time; COVID-19 status did not significantly influence this measure of physical activity.

## 4. Discussion

We found that the level of physical activity as measured through repeated 24 h activity tracking with activPAL3^®^ was low in hospitalised COVID-19 and non-COVID-19 patients. We also found that the measures of sarcopenia, including the SARC-F questionnaire, hand grip strength, and calf circumference, and, to some extent the age-adjusted Charlson comorbidity index, were correlated with the measures of physical activity. Likewise, a greater length of hospital stay was correlated with a worse physical activity level at discharge. Conversely, neither SpO2% nor the severity of acute condition assessed with the MEWS inventory were correlated with physical activity level in these patients.

In the case of the relation between pre-hospitalisation SARC-F and time spent walking at discharge, this was independent of COVID-19 status.

Our findings have several clinical implications. First, they underline the role of prehospitalisation physiological skeletal muscle reserves and multimorbidity in older individuals. The assessment of muscular status during the initial days of hospitalisation, using simple tools such as SARC-F, measurement of calf circumference, and measurement of hand-grip strength may help identify patients who would require more attention from the rehabilitation team. Second, our findings indicate that the imposition of distancing and isolation regimes in a hospital is likely to affect both the patients directly subjected to such measures and those who, in theory, do not require such containment measures. Finally, in both groups mentioned above, ways to improve mobility should be envisaged, irrespective of the patient’s status, so as to ensure optimal functional outcomes.

Several studies from before the COVID-19 pandemic focused on tracking physical activity in older inpatients [14,15]. Theou et al. concluded in their study of 111 patients that, while in hospital, the patients spent only 6% of their waking time in an upright position [16]. Villumsen et al. found that the median walking time of 124 patients hospitalised in the acute geriatric ward was 7 min. Those who were able to perform a timed up-and-go test (TUG) at baseline had the potential for an improvement in physical activity [17]. Lim et al., analysing the data of 38 inpatients, found that the average time spent engaging in physical activity was 4.2 h. However, it is important to note that this time was fragmented into interval of 1–5 min [18]. Pavon et al. found that the average daily time spent walking by 46 community dwelling older inpatients of a general ward was 1.1 h [19]. Our findings confirm and extend these findings. The physical activity of older hospitalised patients is appallingly low. The activity before discharge from the hospital is affected by the length of stay, the degree of comorbidity, and particularly by sarcopenia. We found that the activity was restricted, independent of COVID-19 status. This may be as a result of the personnel’s attitudes and the imposition of COVID-19-related sanitary measures, applicable not only in the COVID-19 ward, but also, to some extent, in non-COVID-19 wards.

Our study should be considered in the context of its limitations. Our group was small and the selection bias cannot be dismissed. Likewise, the observational nature and the lack of an a priori sample size calculation might have led to the high risk of type II errors. As our study was observational, no causality can be assumed. These are factors that may severely hinder the generalizability of our findings. On the one hand, the above-mentioned studies, with methodology similar to ours, were performed in groups of a similar size. We only included eight COVID-19 patients, mostly with a moderate severity disease; however, the implementation of strict protocol requiring repeated use of activPAL3^®^ devices and the muscular assessment by staff using high-grade PPE in an environment dedicated to a high level of antiviral protection made the inclusion of more patients difficult. On the other hand, the very same restrictions and regimes that make research difficult, may have adversely influenced the physical activity in these patients. Our results, although based on a small group of patients, offer several points for consideration during possible future pandemics, as well as in the currently frequent situations that require hospitalisation of individuals with potentially highly infectious respiratory viral infections. First, all efforts should be made to increase the general level of physical activity in older subjects, thus fostering healthy ageing with the potential to prevent or ameliorate sarcopenia. This would improve physical activity at the onset and during hospitalisation. Second, the severity of the disease for which the patient is being hospitalised should not preclude rehabilitation. Third, staff caring for older patients, especially during crises such as the COVID-19 pandemic, when mobility restrictions are imposed, should proactively identify patients at risk of inactivity. Consequently, routines should be established that promote (vigorous) rehabilitation for such patients. Lastly, the guidelines for the type and level of physical activity in older multimorbid hospitalised patients should be established, taking into consideration the patient’s muscular status. However, knowledge gaps still exist, making it necessary to perform more research in the field. All such research should be rigorously designed and conducted through multicentre international studies to facilitate the development of universally adoptable assessment methodologies, including outcomes, and guidelines based on evidence.

## Figures and Tables

**Table 1:** The Fisher method-corrected Spearman correlation analysis of physical activity and patients’ clinical characteristics in 31 patients.

		Stand1	Stand2	Sit/Recl1	Sit/Recl2	Step1	Step2	d_stand	d_sit/recl	d_step
SpO2 admission	r (95% CI) *p*	−0.04 (−0.39, 0.32) 0.82	0.16 (−0.20, 0.49) 0.37	0.07 (−0.29, 0.42) 0.70	−0.13 (−0.46, 0.24) 0.49	0.24 (−0.13−0.53) 0.19	0.21 (−0.15, 0.53) 0.24	−0.17 (−0.49, 0.20) 0.35	0.16 (−0.21, 0.48) 0.40	−0.01 (−0.37, 0.34) 0.94
SARC_F	r (95% CI) *p*	−0.59 (−0.78, −0.29) 0.0003	−0.58 (−0.77, −0.28) 0.0004	0.57 (0.27, 0.77) 0.0005	0.61 (0.32, 0.79) 0.0002	−0.52 (−0.74, −0.21) 0.002	−0.66 (−0.82, −0.40) <0.001	0.23 (−0.13, 0.54) 0.20	−0.21 (−0.53, 0.16) 0.25	0.37 (0.02, 0.64) 0.03
MEWS	r (95% CI) *p*	0.09 (−0.28, 0.43) 0.64	0.16 (−0.21, 0.49) 0.39	−0.08 (−0.42, 0.28) 0.68	−0.19 (−0.50, 0.18) 0.32	−0.01 (−0.37, 0.34) 0.94	0.20 (−0.16, 0.52) 0.27	−0.11 (−0.44, 0.26) 0.56	0.13 (−0.23, 0.46) 0.47	−0.20 (−0.52, 0.16) 0.26
aCCI	r (95% CI) *p*	−0.33 (−0.61, 0.03) 0.07	−0.43 (−0.68, −0.09) 0.01	0.35 (0.00, 0.63) 0.05	0.45 (0.11, 0.69) 0.009	−0.20 (−0.52, 0.17) 0.28	−0.34 (−0.62, 0.02) 0.06	0.11 (−0.26, 0.44) 0.57	−0.11 (−0.45, 0.25) 0.55	0.18 (−0.19, 0.50) 0.33
LoS	r (95% CI) *p*	… …	−0.52 (−0.74, −0.21) 0.002	… …	0.54 (0.23, 0.75) 0.001	… …	−0.51 (−0.73, −0.19) 0.003	… …	… …	… …
HGR admission	r (95% CI) *p*	0.53 (0.21, 0.74) 0.002	0.49 (0.17, 0.72) 0.004	−0.55 (−0.76, −0.24) 0.0009	−0.51 (−0.73, −0.20) 0.002	0.47 (0.14, 0.71) 0.006	0.59 (0.30, 0.78) 0.0002	−0.10 (−0.44, 0.26) 0.58	0.05 (−0.31, 0.40) 0.78	−0.33 (−0.61, 0.03) 0.07
CC admission	r (95% CI) *p*	0.36 (0.00, 0.64) 0.04	0.53 (0.22, 0.74) 0.002	−0.38 (−0.65, −0.03) 0.03	−0.54 (−0.75, −0.23) 0.001	0.42 (0.07, 0.62) 0.02	0.44 (0.10, 0.69) 0.01	−0.15 (−0.48, 0.22) 0.43	0.13 (−0.24, 0.46) 0.50	−0.09 (−0.43, 0.27) 0.63

r—correlation coefficient (estimate); 95%CI—95% confidence interval; *p*—*p* value; Stand1, 2—time spent standing upon admission (1) and at discharge (2), respectively; Sit/Recl 1, 2—time spent sitting or reclining; Step 1, 2—time spent walking; d_—difference between upon admission and at discharge. SpO2—blood oxygen saturation; SARC-F—sarcopenia screening tool; MEWS—Modified Early Warning Score; aCCI—Charlson Index; LoS—length of hospital stay; HGR—handgrip strength; CC—calf circumference.

## Data Availability

Upon reasonable request.

## References

[1] Tasheva P., Vollenweider P., Kraege V., Roulet G., Lamy O., Marques-Vidal P., Méan M. (2020). Association Between Physical Activity Levels in the Hospital Setting and Hospital-Acquired Functional Decline in Elderly Patients. JAMA Netw. Open.

[2] Pedersen M.M., Bodilsen A.C., Petersen J., Beyer N., Andersen O., Lawson-Smith L., Kehlet H., Bandholm T. (2013). Twenty-four-hour mobility during acute hospitalization in older medical patients. J. Gerontol. A Biol. Sci. Med. Sci..

[3] Lim S.E.R., Ibrahim K., Sayer A.A., Roberts H.C. (2018). Assessment of Physical Activity of Hospitalised Older Adults: A Systematic Review. J. Nutr. Health Aging.

[4] Piotrowicz K., Gąsowski J., Michel J.-P., Veronese N. (2021). Post-COVID-19 acute sarcopenia: Physiopathology and management. Aging Clin. Exp. Res..

[5] Kosse N.M., Dutmer A.L., Dasenbrock L., Bauer J.M., Lamoth C.J.C. (2013). Effectiveness and feasibility of early physical rehabilitation programs for geriatric hospitalized patients: A systematic review. BMC Geriatr..

[6] Heldmann P., Werner C., Belala N., Bauer J.M., Hauer K. (2019). Early inpatient rehabilitation for acutely hospitalized older patients: A systematic review of outcome measures. BMC Geriatr..

[7] De Spiegeleer A., Kahya H., Sanchez-Rodriguez D., Piotrowicz K., Surquin M., Marco E., Detremerie C., Hussein D., Hope S., Dallmeier D. (2021). Acute sarcopenia changes following hospitalization: Influence of pre-admission care dependency level. Age Ageing.

[8] Grund S., Gordon A.L., van Balen R., Bachmann S., Cherubini A., Landi F., Stuck A.E., Becker C., Achterberg W.P., Bauer J.M. (2020). European consensus on core principles and future priorities for geriatric rehabilita-tion: Consensus statement. Eur. Geriatr. Med..

[9] Piotrowicz K., Głuszewska A., Czesak J., Fedyk-Łukasik M., Klimek E., Sánchez-Rodríguez D., Skalska A., Gryglewsaka B., Grodzicki T., Gąsowski J. (2021). SARC-F as a case-finding tool for sarcopenia according to the EWGSOP2. National validation and comparison with other diagnostic standards. Aging Clin. Exp. Res..

[10] Sundararajan V., Henderson T., Perry C., Muggivan A., Quan H., Ghali W.A. (2004). New ICD-10 version of the Charlson comorbidity index predicted in-hospital mortality. J. Clin. Epidemiol..

[11] Subbe C.P., Kruger M., Rutherford P., Gemmel L. (2001). Validation of a modified Early Warning Score in medical admissions. QJM.

[12] Cruz-Jentoft A.J., Bahat G., Bauer J., Boirie Y., Bruyère O., Cederholm T., Cooper C., Landi F., Rolland Y., Sayer A.A. (2019). Sarcopenia: Revised European consensus on definition and diagnosis. Age Ageing.

[13] CDC (2020). Healthcare Workers. Centers for Disease Control and Prevention. https://www.cdc.gov/coronavirus/2019-ncov/hcp/infection-control.html.

[14] Baldwin C., van Kessel G., Phillips A., Johnston K. (2017). Accelerometry Shows Inpatients With Acute Medical or Surgical Conditions Spend Little Time Upright and Are Highly Sedentary: Systematic Review. Phys. Ther..

[15] Chan C.S., Slaughter S.E., Jones C.A., Ickert C., Wagg A.S. (2017). Measuring Activity Performance of Older Adults Using the activPAL: A Rapid Review. Healthcare.

[16] Theou O., Kehler D.S., Godin J., Mallery K., MacLean M.A., Rockwood K. (2019). Upright time during hospitalization for older inpatients: A prospective cohort study. Exp. Gerontol..

[17] Villumsen M., Jorgensen M.G., Andreasen J., Rathleff M.S., Mølgaard C.M. (2015). Very Low Levels of Physical Activity in Older Patients During Hospitalization at an Acute Geriatric Ward: A Prospective Cohort Study. J. Aging Phys. Act..

[18] Lim S.E.R., Dodds R., Bacon D., Sayer A.A., Roberts H.C. (2018). Physical activity among hospitalised older people: Insights from upper and lower limb accelerometry. Aging Clin. Exp. Res..

[19] Pavon J.M., Sloane R.J., Pieper C.F., Colón-Emeric C.S., Cohen H.J., Gallagher D., Morey M.C., McCarty M., Hastings S.N. (2020). Accelerometer-Measured Hospital Physical Activity and Hospital-Acquired Disability in Older Adults. J. Am. Geriatr. Soc..

